# Supplementary material for: Exploring the Genetic Networks of HLB Tolerance in Citrus: Insights Across Species and Tissues
Source: Plants (Basel). 2025 Jun 11;14(12):1792. doi: 10.3390/plants14121792 (PMC12197043; doi:10.3390/plants14121792)
Supplement: Supplementary file 1 [file plants-14-01792-s001.zip › Supplementary Figures.pdf]

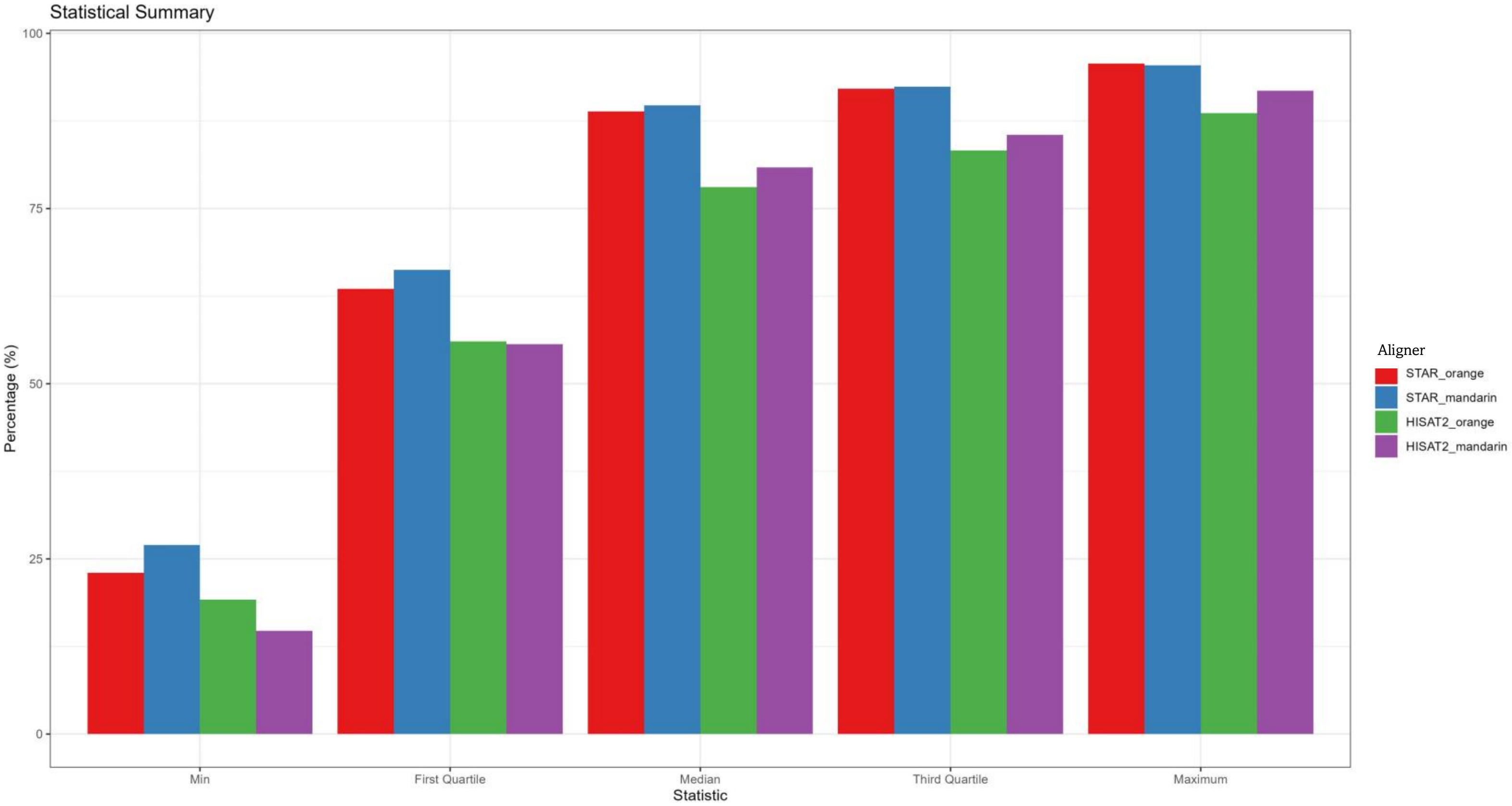

Figure S1: Statistical summary of mapping performance (STAR vs. HISAT2)

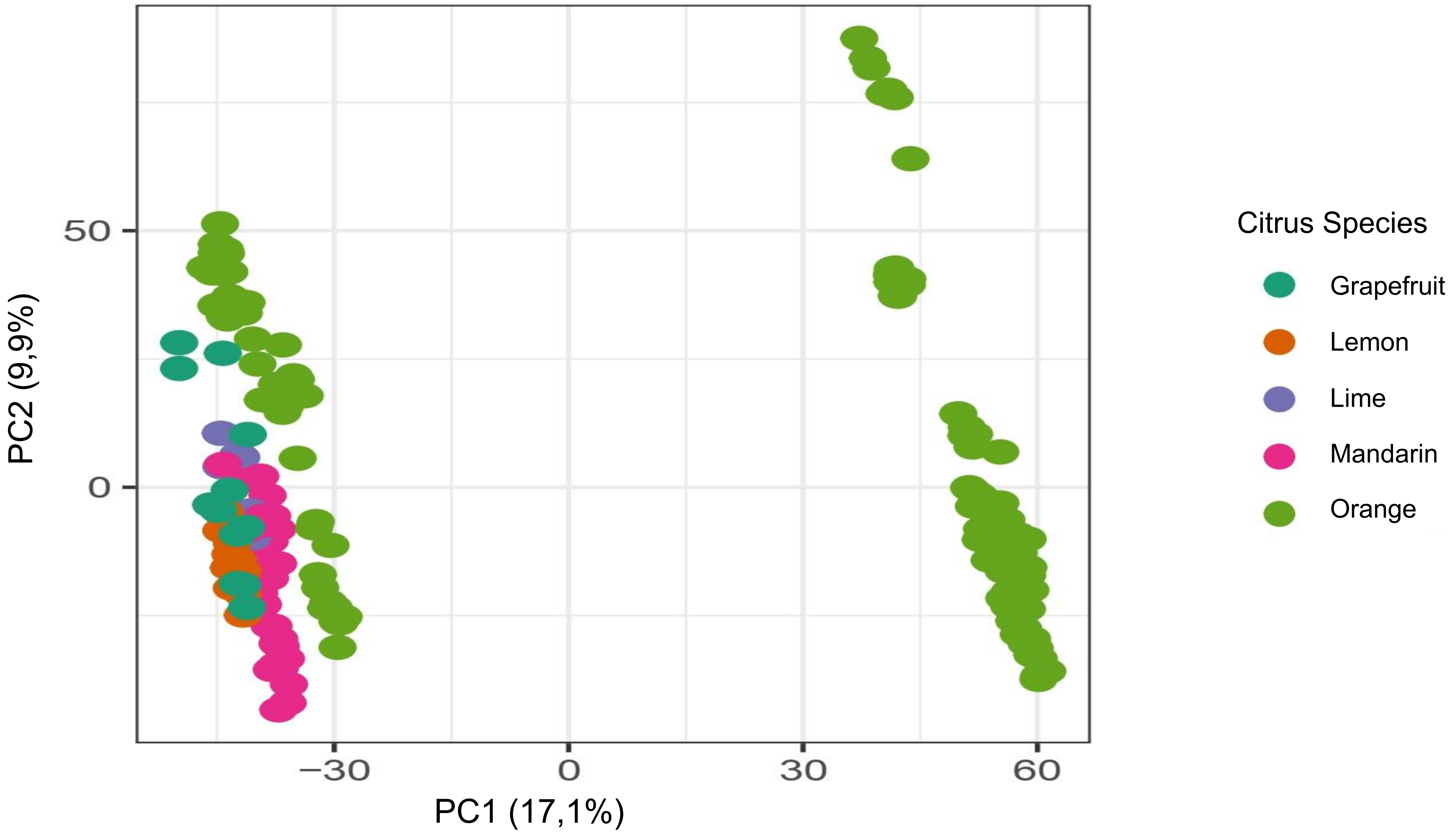

Figure S2-A. PCA plot shows distinct clustering of 230 RNA-seq samples by *Citrus species*.

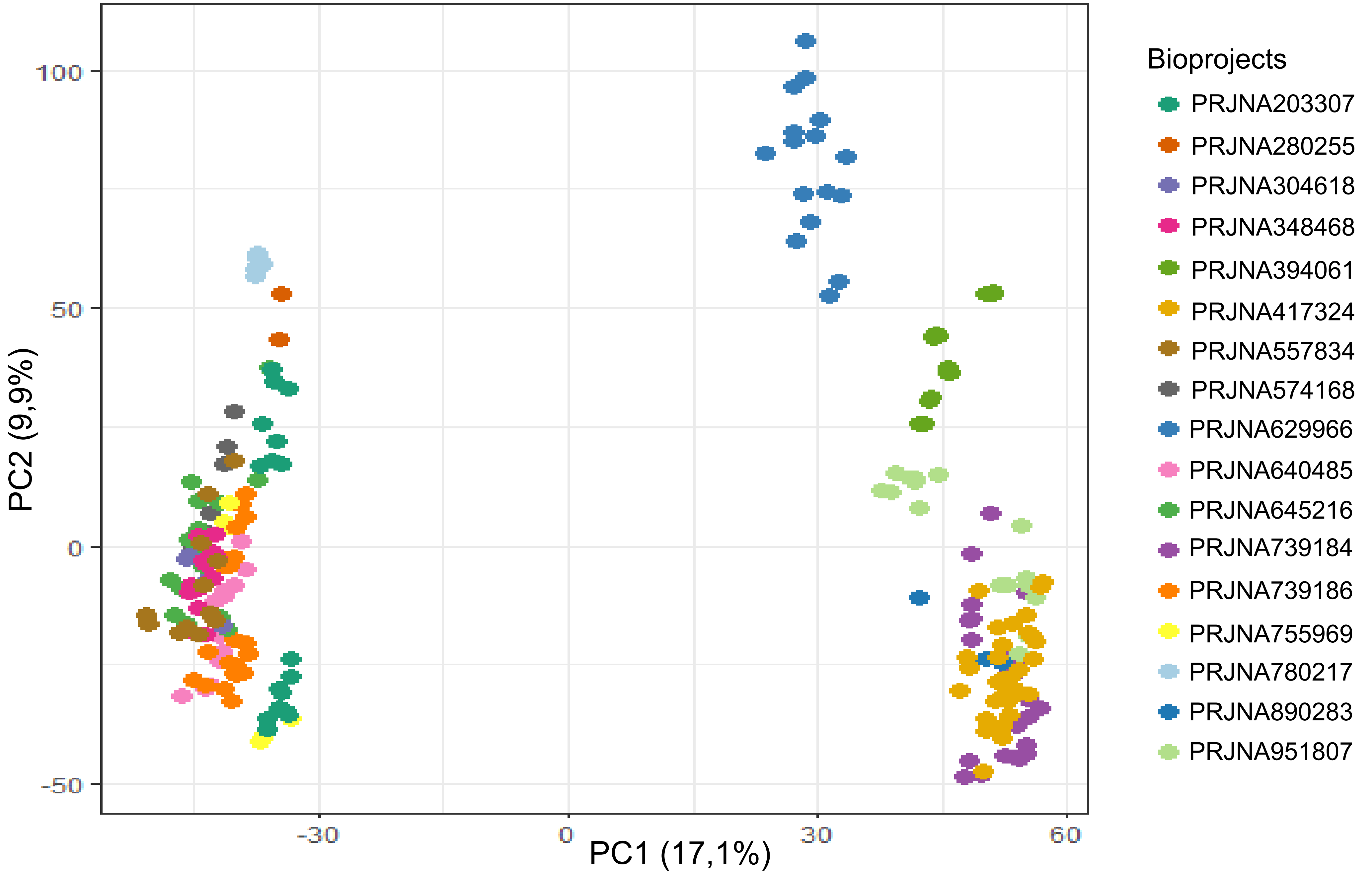

Figure S2-B. PCA plot shows distinct clustering of 230 RNA-seq samples by *Bioprojects*

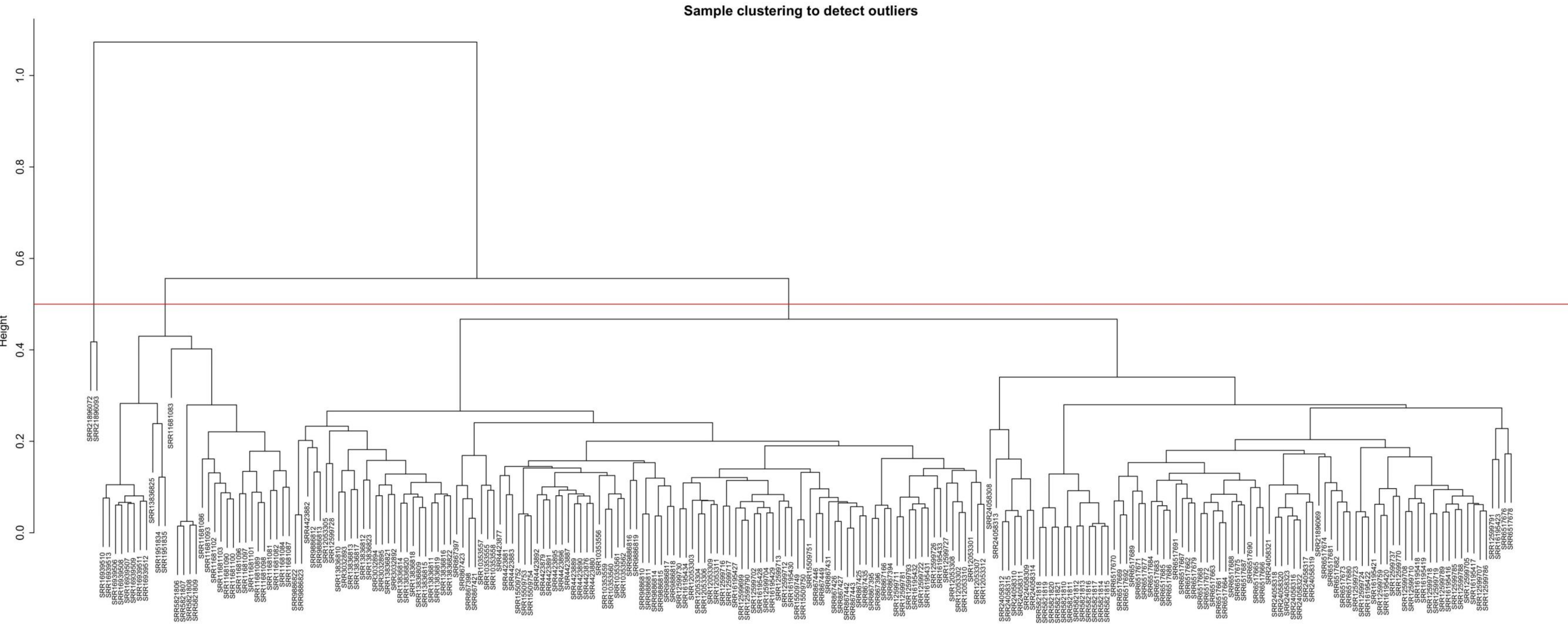

Figure S3. Hierarchical clustering dendrogram of gene expression samples based on Pearson correlation. A height cutoff of 0.5 identified 197 retained samples (Cluster 1) and excluded 33 outliers.

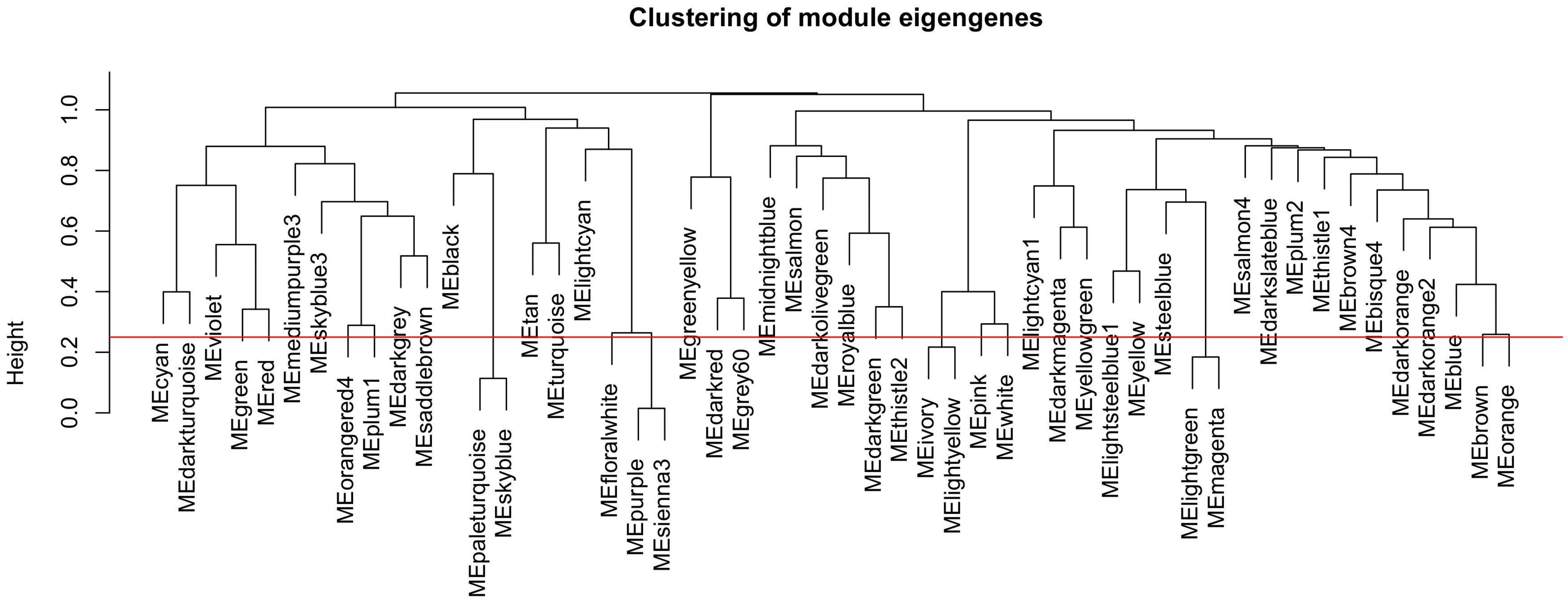

Figure S4. Hierarchical clustering of module eigengenes based on their correlation, where a red horizontal line signifies the chosen cut-off height (dissimilarity of 0.25) for merging modules with eigengene correlations exceeding 0.75.

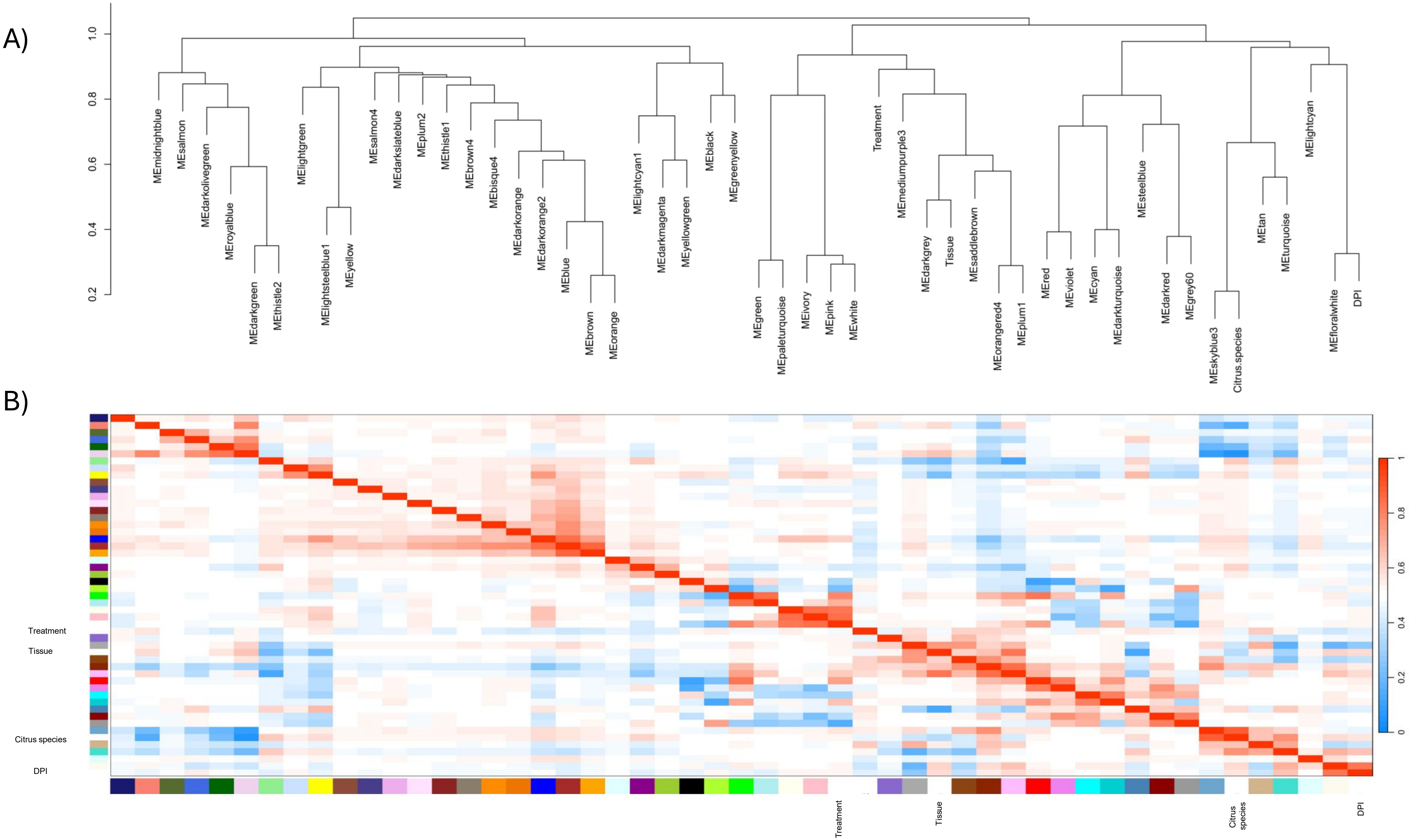

Figure S5. Eigengene Network and Module-Trait Relationships. (A) Dendrogram of module eigengene clustering. (B) Heatmap of module eigengene adjacency, with color-coded boxes indicating correlation strength. Traits (citrus species, DPI, tissue type and treatment) are integrated to visualize their relationships with the module eigengenes.

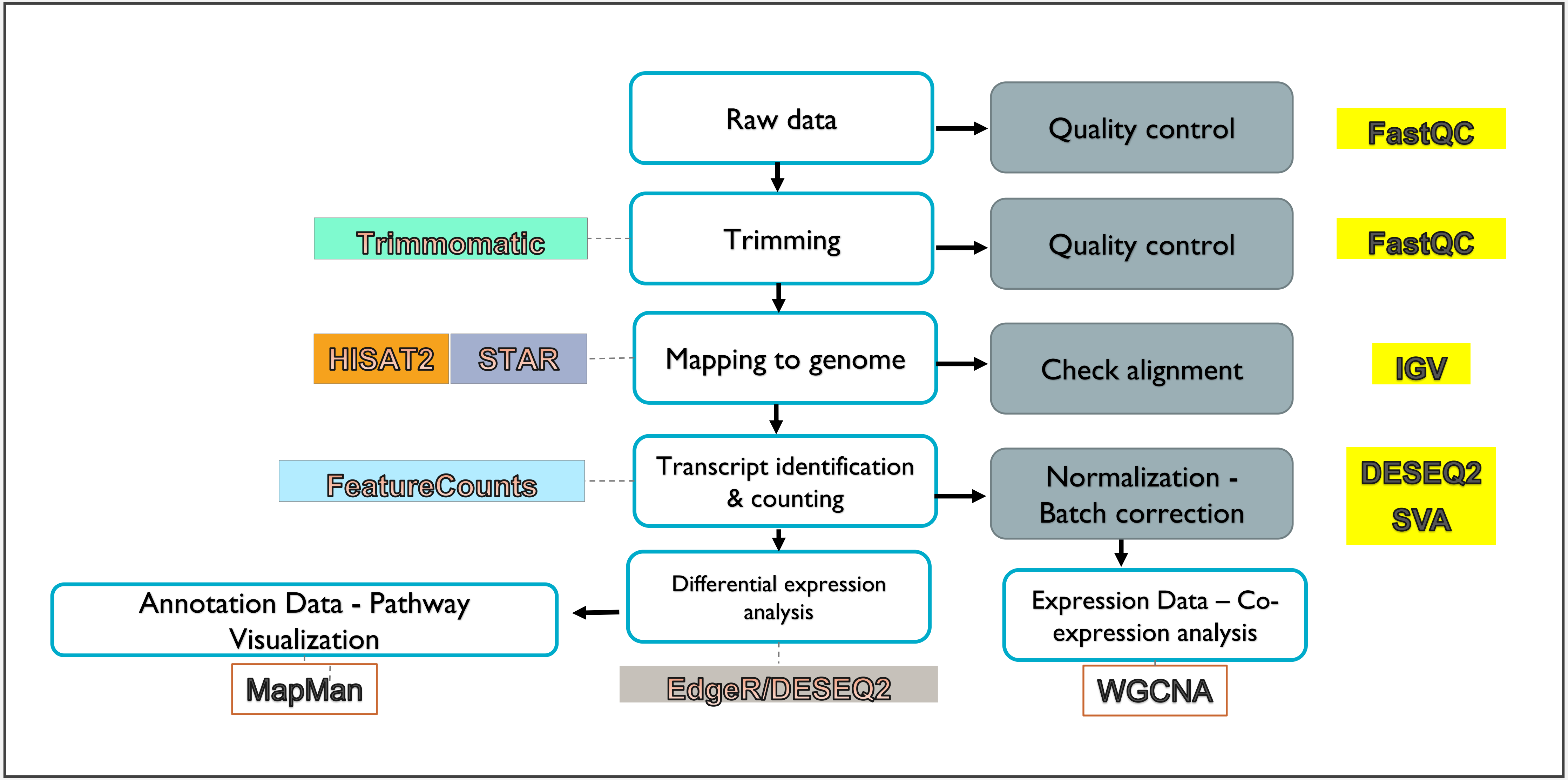

Figure S6. Bioinformatic workflow describing sample processing for co-expression network analysis and differential expression analysis (DEGs)
